# Topological phases and non-Hermitian topology in photonic artificial microstructures

**DOI:** 10.1515/nanoph-2022-0778

**Published:** 2023-02-16

**Authors:** Hui Liu, Pengtao Lai, Haonan Wang, Hua Cheng, Jianguo Tian, Shuqi Chen

**Affiliations:** The Key Laboratory of Weak Light Nonlinear Photonics, Ministry of Education, Smart Sensing Interdisciplinary Science Center, Renewable Energy Conversion and Storage Center, School of Physics and TEDA Institute of Applied Physics, Nankai University, Tianjin 300071, China; The Collaborative Innovation Center of Extreme Optics, Shanxi University, Taiyuan, Shanxi 030006, China

**Keywords:** non-Hermitian topology, topological insulator, topological photonics, topological semimetal

## Abstract

In the past few decades, the discovery of topological matter states has ushered in a new era in topological physics, providing a robust framework for strategically controlling the transport of particles or waves. Topological photonics, in particular, has sparked considerable research due to its ability to construct and manipulate photonic topological states via photonic artificial microstructures. Although the concept of topology originates from condensed matter, topological photonics has given rise to new fundamental ideas and a range of potential applications that may lead to revolutionary technologies. Here, we review recent developments in topological photonics, with a focus on the realization and application of several emerging research areas in photonic artificial microstructures. We highlight the research trend, spanning from the photonic counterpart of topological insulator phases, through topological semimetal phases, to other emerging non-Hermitian topologies.

## 1 Introduction

A deep understanding of topological matter states gives topology new physical meaning, opening the chapter of topological physics. The origin of topological physics dates back to the discovery of the integer quantum Hall effect (IQHE) in condensed matter physics [1]. The robust quantized Hall conductance observed in a two-dimensional (2D) electron gas arises from a nonzero topological invariant, well known as the Chern number. An important insight into the physical meaning of topological invariant is the proposal of the bulk-edge correspondence [2]. Topologically protected boundary states appear on the interface of topological materials with different topological invariants [3]. These topologically protected states are unprecedented ideal transport states, and they have the potential to revolutionize traditional technologies and devices. The exploration of topological phases and the relevant topological phenomena is in full swing in broad areas of physics, including photonics [4–7], phononics [8–10], mechanics [11–13], and cold atoms [14, 15]. Among them, topological photonics has drawn great interest, not only for the advantages of optical materials in constructing topological phases but also for the urgent need for robust properties in optical communication and transmission.

Since artificial microstructures, including metamaterials and photonic crystals, provide great possibilities for manipulating optical waves, a series of novel phenomena and functionalities are generated, such as negative refractive index [16], invisibility cloaks [17], and chiral optical response [18]. In this context, optical systems are considered as a potential platform for topological physics research. The photonic topological insulator, transplanted from condensed matter, requires overcoming the natural difference between bosons and fermions. Considerable efforts have been made to construct Kramers degeneracy and realize fundamental topological models of topological insulators in optical systems, giving rise to the realization of photonic IQHE [19–21], photonic quantum spin Hall effect (QSHE) [22, 23], photonic quantum valley Hall effect [24], and photonic Floquet topological insulators [25, 26]. Thus, topology, serving as a new concept, characterizes the quantized global behavior of wave functions in optics, providing a robust way to control and transport optical waves in waveguides, lasers, and optical devices. Owing to the clean energy band and flexible ability to control it, topological photonics play an important role in exploring novel topological phases and phenomena. With the development of research and experimental techniques, the exploration of topological phases has been extended to higher dimensions, including three dimensions (3D) [27–29] and four dimensions (4D) [30]. Among the 3D topological phases, topological semimetals [31] have drawn great attention, where stable degeneracies with nonzero topological charges are connected by topologically protected surface states known as Fermi arcs. Moreover, the discovery of novel topological phases, including 3D and 4D topological insulators [30, 32] and higher-order insulators [23, 33] in optical systems, provides new insights into the fundamental meanings of topological matter states. Recently, the concept of topology further penetrated into optical nonlinear [34–36], non-Hermitian [37–39], non-Abelian [40–42], and disorder systems [43, 44], inspiring considerable research and application. In particular, non-Hermitian topology is constructed by loss that is not welcome in optical systems, showing novel topological properties and great application potential.

In this review, we follow the fast progress in topological photonics. This review is divided into three parts, where the realization and application of photonic topological phases are introduced in photonic crystals or metamaterials. In the first part, we introduce the category of 2D photonic topological insulators described by distinct topological invariants, including the quantum Hall, quantum spin Hall, and quantum valley Hall effects. In the second part, extending to 3D space, we discuss the realization of photonic topological semimetals. The exploration of distinct 3D degeneracies, including Weyl points, 3D Dirac points, nodal lines, and surfaces, and the relevant novel topological surface states are presented. In the last part, we introduce the non-Hermitian topology, characterized by exceptional degeneracies, and non-Hermitian skin effects that break through the traditional bulk-edge correspondence and revise our understanding of band topology.

## 2 2D photonic topological insulator

### 2.1 Photonic integer quantum Hall effect

IQHE was discovered by K. V. Klitzing et al. in 1980 [1]. A 2D electron gas presented a robust quantization of the Hall conductance by adding a strong magnetic field. Further research found that the quantization of the Hall conductance can be related to the number of unidirectional edge states, which were determined by the bulk topological invariant, known as the Chern number. The relationship between the number of edge states and the bulk topological invariant is well known as the bulk-edge correspondence. The Chern number, describing the quantized global behavior of wave functions in one bulk band, can be defined as:

(1) $$C_{n}=\frac{1}{2\pi }\underset{BZ}{\int \int }\Omega_{n}\left(k\right)d^{2}k=\frac{1}{2\pi }\underset{BZ}{\int \int }\nabla_{k}\times A_{n}\left(k\right)dk$$

where Ω_*n*_(*k*) is the Berry curvature for the *n*th energy band, $A_{n}\left(k\right)=i\left\langle u_{n,k}\right|\nabla_{k}\left|u_{n,k}\right\rangle$ is the Berry connection and *u*_*n*_(*k*) indicates the periodic part of the Bloch function of the *n*th eigenstate. Under a gauge transformation *u*_*n*_(*k*) → *u*_*n*_(*k*)e^i*φ*(*k*)^, the Berry connection is not gauge invariant and transforms as *A*_*n*_(*k*) → *A*_*n*_(*k*) − ∇_*k*_ *φ*(*k*). As the curl of the divergence is zero, the Berry curvature *F*(*k*) = ∇_*k*_ × *A*_*n*_(*k*) is gauge invariant. The Berry connection and Berry curvature are similar to the vector potential *A*(*r*) and magnetic field B, where the magnetic field B is gauge invariant under the gauge transformation of *A*(*r*) → *A*(*r*) + ∇*φ*(*r*). Thus, the Chern number, which is the integration of Berry curvature over the first Brillouin zone (BZ), can be viewed as the number of monopoles of Berry flux inside a closed surface. This comparison gives a clearer picture of the topological charge and may help the understanding of the Weyl point in Section 3.

**Figure 1:**
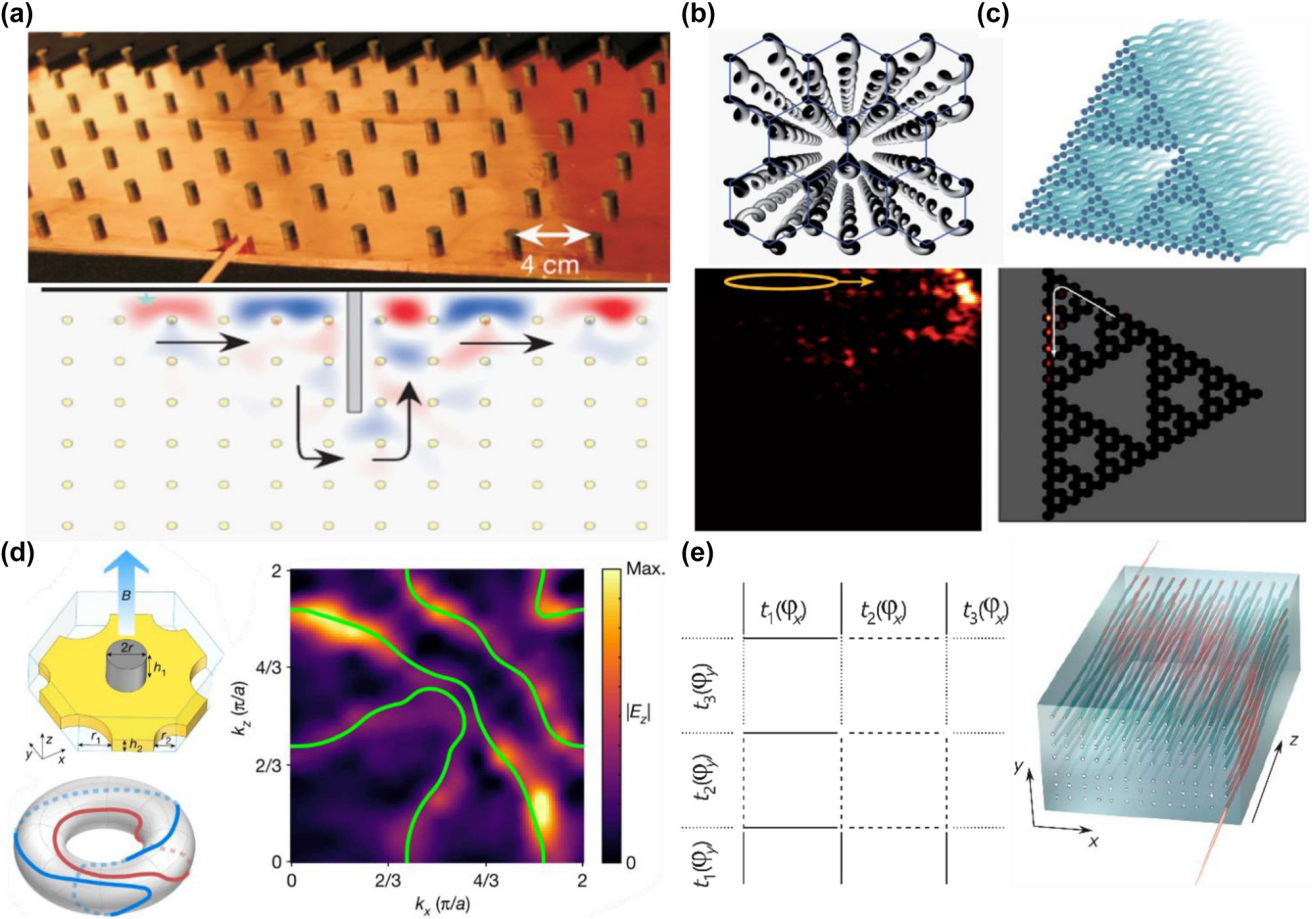
Analog quantum Hall effect in photonics. (a) Experimental observation of photonic IQHE and the unidirectional waveguide in gyromagnetic photonic crystal [21]. (b) Schematic of an array of helical waveguides in the hexagonal lattice and the time evolution of the input light [25]. (c) Photonic fractal lattice and experimental observation of the photonic quantum Hall effect [51]. (d) Topological Chern vectors in the three-dimensional gyromagnetic photonic crystal (left) and Hopf link surface states in the surface BZ (right) [11]. (e) The coupling and waveguides of 4D quantum Hall effect [30].

In photonic systems, Haldane et al. proposed that the IQHE can be achieved in the gyroelectric photonic crystal [19]. A pair of Dirac points can be constructed by the transverse electric (TE) photonic bands in the hexagonal crystal, and then an external magnetic field is added to break it. In realistic gyroelectric materials, because the ratio of off-diagonal to diagonal elements of the permittivity tensor is small, the strength of time reversal breaking is weak [20]. Thus, Wang et al. used the gyromagnetic material yttrium-iron-garnet (YIG) to experimentally verify the photonic quantum Hall effect [21]. They demonstrated the existence of a unidirectional edge state in the square lattice, as shown in Figure 1a. In the gyromagnetic photonic crystal, band structures with arbitrary Chern numbers can be generated in two steps. First, an arbitrary number of Dirac points are generated in the first BZ by the band folding mechanism. Then, open the multiple Dirac points simultaneously by applying a static magnetic field [29, 45], [46], [47]. Based on the magneto-optical effect in gyromagnetic photonic crystals, novel devices such as unidirectional waveguides [48, 49] and topological lasers [50] can be designed.

Because the magneto-optical responses of materials are weak and it is difficult to cover the light wavelength, another method of realizing the quantum Hall effect based on the propagation system [25] is proposed, namely, Floquet topological insulators. Paraxial propagation of light in photonic lattices is described by the Schrodinger-type equation:

(2) $$\begin{array}{ll} i\partial_{z}\Psi \left(x,y,z\right) & =-\frac{1}{2k_{0}}\nabla^{2}\Psi \left(x,y,z\right)\\ & \;-\frac{k_{0}\Delta n\left(x,y,z\right)}{n_{0}}\Psi \left(x,y,z\right) \end{array}$$

where Ψ(*x*, *y*, *z*) is the electric field envelope function, *k*_0_ = 2*πn*_0_/*λ* is the wavenumber in the ambient medium, *n*_0_ represents the refractive index of the ambient medium, and Δ*n* is the deviation from the ambient refractive index. In the paraxial propagation equation, the propagation coordinate *z* takes the role of time. Thus, breaking *z* reversal symmetry can mimic the breaking of time reversal (*T*) symmetry in 2D systems. The helical waveguides induce a gauge vector potential *A*(*z*) = *kR*Ω(sin Ω*z*, −cos Ω*z*, 0), where *R* and Ω represent the radius and the longitudinal frequency in the helix, respectively, as shown in Figure 1b. The relevant topological protected edge state is shown in the lower panel of Figure 1b. Very recently, T. Biesenthal et al. experimentally demonstrated the existence of the quantum Hall effect in the fractal lattice [51]. Evanescently coupled helical waveguides with a Hausdorff dimension of 1.585 [51, 52] are arranged as a Sierpinski gasket (SG), as shown in Figure 1c. As shown in the lower panel of Figure 1c, although SG lacks the conventional bulk and is in a noninteger dimension, there is a topological protected edge state traveling around the sample. Since the fractal lattice is nonperiodic, the topology of the system can be characterized by the real space Chern number [53, 54].

Recently, higher dimensional Chern insulators, as an extension of 2D Chern insulators, have been constructed in photonic crystals. In contrast to the 2D Chern insulator, the 3D Chern insulator can be characterized by the Chern vector *C* = (*C*_*x*_, *C*_*y*_, *C*_*z*_), where *C*_*x*_, *C*_*y*_, and *C*_*z*_ are three first Chern numbers in lower dimensional surfaces [47]. Liu et al. experimentally constructed a 3D Chern vector insulator, where the *T* symmetry is broken by a gyromagnetic material [29], as shown in Figure 1d. Due to the multiple components of the Chern vector, there is a generalized bulk-edge correspondence. The relevant intersurface states can map to nontrivial torus knots (also called Hopf links) on the surface BZ, as shown in the right panel of Figure 1c.

In 4D systems, the quantum Hall effect can be characterized by the second Chern number [30, 55, 56], which can be defined as [30]:

(3) $$C_{2}=-\frac{1}{8\pi^{2}}\int \varepsilon_{\mu \nu \rho \sigma }tr\left\{P_{j}\frac{\partial P_{j}}{\partial k_{\mu }}\frac{\partial P_{j}}{\partial k_{\nu }}P_{j}\frac{\partial P_{j}}{\partial k_{\rho }}\frac{\partial P_{j}}{\partial k_{\sigma }}\right\}d^{4}k$$

*P*_*j*_(*k*) is the projector onto the subspace spanned by the eigenstates with energies below the bandgap. As sketched in Figure 1d, the 4D quantum Hall system can be decomposed into two independent one-dimensional (1D) topological pumps [30]. Each 1D topological pump is topologically equivalent to a 2D Harper model [57], which can be characterized by the first Chern number. Thus, the eigenstates of the full Hamiltonian are the tensor product of the eigenstates in the two independent 1D topological pumps, and the spectrum of the full Hamiltonian is a Minkowski sum of the spectra of the two 1D pumps. The second Chern number can also be calculated as the product of the first Chern number [30].

### 2.2 Photonic quantum spin Hall effect

The QSHE was proposed by C. L. Kane et al. [58, 59] and B. A. Bernevig et al. [60, 61] in graphene and HgTe quantum wells, respectively. Subsequently, the QSHE was experimentally realized in HgTe quantum wells [62]. The QSHE can be regarded as two copies of the quantum Hall effect that each represents a spin, and the subspace for each spin is related by *T* symmetry. There are two helical edge states in the QSHE, and the group velocity of each helical edge state is locked by the spin. The spin Chern number in the QSHE can be defined as *C*_spin_ = (*C*_up_ − *C*_down_)/2 (mod 2), where *C*_up_ is the Chern number of spin up and *C*_down_ is the Chern number of spin down. Protected by *T* symmetry, the total Chern number (*C*_total_ = *C*_up_ + *C*_down_) in the systems always equals to zero. In this case, the topology of the QSHE is captured by *C*_spin_, which is equivalent to the *Z*_2_ topological invariant [63, 64]. In the *Z*_2_ topological invariant, “0” represents trivial and “1” represents nontrivial. In 3D, the topological phases for *T* invariant insulators are classified by four *Z*_2_ topological invariants $\nu_{0}\left(\nu_{1},\nu_{2},\nu_{3}\right)$ , where *ν*_1_, *ν*_2_, and *ν*_3_ are the *Z*_2_ topological invariants in the *k*_*x*_ = *π*, *k*_*y*_ = *π*, and *k*_*z*_ = *π* planes, respectively, and *ν*_0_ is the product of the *Z*_2_ topological invariants in the *k*_*x*_ = 0 and *k*_*x*_ = *π* planes [32, 65]. The topological phases can be classified into three distinct classes by $\nu_{0}\left(\nu_{1},\nu_{2},\nu_{3}\right)$ in 3D. For *ν*_0,1,2,3_ = 0, these phases are trivial. For *ν*_0_ = 0, *ν*_1,2,3_ ≠ 0, there exists delicate surface states, which can be broken by disorders, thus these phases are dubbed weak topological insulator. The weak topological insulator can be interpreted as the stacking of 2D QSHE. The *ν*_0_ = 1 phases are more robust and are referred to as strong topological insulator. From the retrospective of the introduction in IQHE and QSHE, it is easy to draw the conclusion that the spatial dimension is one of the decisive parameters for the classification of topological phase.

**Figure 2:**
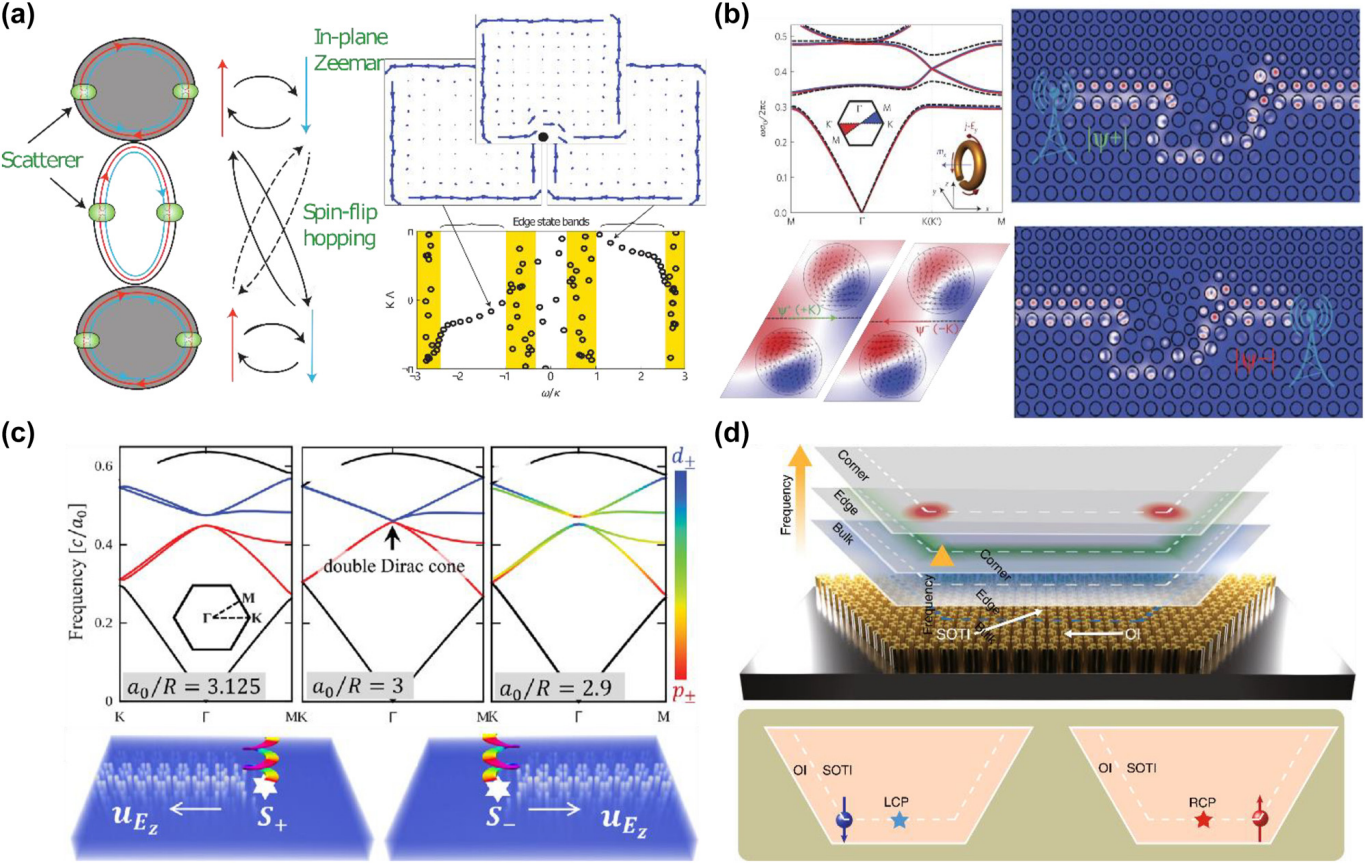
Analog quantum spin Hall effect in photonics. (a) Schematic of two coupled resonators (left). Topological edge state (top-right) and their energy dispersion for single spin (bottom-right) [22]. (b) Photonic QSHE based on bianisotropic metamaterials [69]. Left: The bulk band structure and the eigenmodes near the point degeneracy. Right: Pseudospin-dependence edge states [69]. (c) Scheme for achieving an analog of the QSHE in the hexagonal lattice [74]. (d) High-order QSHE in the hexagonal lattice [23].

In photonics, the QSHE has been achieved by M. Hafezi et al. in arrays of coupled resonator optical waveguides [22]. There are a pair of degenerate modes in the resonator, where the clockwise and counterclockwise modes are time-reversed pairs, mimicking the spins in the electronic system, as sketched in Figure 2a. For the counterclockwise mode (pseudospin up), photons acquire different phases between hopping forward and hopping backward. Thus, photons can accumulate a 2*πα* phase around each square plaquette by tuning the phase of the connecting hopping. The pseudospin up (down) component will experience a magnetic field of *α* (−*α*). Moreover, adding scatters can mix the spin, which is similar to the Rashba coupling in the electronic system. The edge state for the counterclockwise mode is shown in the right panel of Figure 2a, while the clockwise mode is reversed by the *T* symmetry, propagating in the opposite direction. In 2013, M. Hafezi et al. experimentally verified the topological edge states and their robustness against defects [66]. Subsequently, they measured the topological invariants in photonic systems [67, 68].

Metamaterials offer a flexible platform to modulate the effective dielectric permittivity *ε*, magnetic permeability *μ*, and bianisotropy *χ*. TM/TE waves have nonvanishing electric/magnetic components in the propagation direction. Another scheme has been proposed to construct a pair of pseudospins by two linear combinations of TM and TE modes *ψ* ^±^ = TE ± TM. For a system in the absence of bianisotropy, when the parameters satisfy *ɛ* = *μ*, the system restores the electromagnetic duality (*E*, *H*) → (−*H*, *E*). It can be proven that a symmetry operator $\hat{D}$ satisfying $\hat{D}\psi^{\pm }=\psi^{\mp }$ and ${\hat{D}}^{2}=-1$ can be identified as pseudo-time reversal symmetry. Nonzero bianisotropy *χ* can mimic the spin–orbit coupling in the electronic system and open a nontrivial gap, as shown in Figure 2b. The bianisotropy in natural materials is usually small, while chiral metamaterials can generate giant bianisotropy [69]. The pseudospin-dependent edge states have been experimentally observed by W. J. Chen et al. in the microwave frequency range [70]. Furthermore, all dielectric metamaterials can also be utilized to generate the QSHE [71, 72]. By shifting the metallic collars from up to down, the effective mass “m” changes the sign. Once the position of the metallic collar can be easily moved, arbitrary and reconfigurable topological domain walls can be designed, which can route and manipulate the propagation of electromagnetic waves [73].

Wu and Hu proposed another scheme to realize the QSHE by *C*_6_ crystal symmetry in photonic systems [74]. There are a pair of pseudospin locked edge states, which propagate in the opposite directions. Under the near field scanning, S. Arora experimentally observed the spin density distribution of topological edge state in graphenelike photonics. Unlike in the electronic system, the spin of edge state in photonic system no longer retains its unique handedness [75]. In Figure 2c, a fourfold degeneracy Dirac point is constructed at the Γ point by band folding. The fourfold degeneracy Dirac point can be gapped by stretching or compressing the interstitial region among artificial atoms, resulting in a trivial or nontrivial bandgap. For the structure with a topological phase, the *k* ⋅ *P* model Hamiltonian around the Γ point is analogous to the Bernevig–Hughes–Zhang model in the HgTe quantum well [60], which implies that the behavior of the *C*_6_ crystal symmetry system is the same as that in the QSHE. This scheme is also wildly applied to realize topological all-optical logic gate [76], topological laser [77–79], and reconfigurable topological insulator [80–82]. However, it should be noted that the topology in this system is protected by the crystalline symmetry, rather than *T* symmetry, and any disorders that break the *C*_6_ symmetry would mix the two pseudospins. In fact, the topology in the *C*_6_ crystal symmetry is fragile [83, 84]. When the edge state is gapped, higher-order topological states (corner states) are generated in the mid-gap [85, 86]. The corner states are also pseudospin locked, thus they can be selectively excited by a source field [23], as shown in Figure 2d.

### 2.3 Photonic valley Hall effect

Valleytronics is a novel research area for designing the degree of valley. Valley is referred to as the energy extrema in reciprocal space, which often emerges at the high symmetry point in the BZ. Due to the large mismatch in momentum, the intervalley scattering is negligibly weak. Thus, valley is a new degree of freedom for the transmission of information and energy.

Photonic valley Hall insulators are usually constructed in 2D hexagonal crystals, such as graphene, with pairs of Dirac cones at two inequivalent corners *K* and *K*′ in the BZ. If the *T* symmetry is broken, similar to the Haldane model, a quantum Hall phase characterized by the Chern number can be induced. If the inversion symmetry or the mirror symmetry is broken while the *T* symmetry is preserved, a valley Hall phase can be induced. Similarly, one can define the integration of Berry curvature in the vicinity of two valleys *K* and *K*′ as the valley Chern number $C_{K/K^{\prime }}$ .

In the presence of *T* symmetry, the Berry curvature is constrained by the relation Ω(−*k*) = −Ω(*k*), where Ω(*k*) is the Berry curvature at momentum *k*, indicating that the Chern number is always zero. Since valleys *K* and *K*′ are related by the *T* symmetry, the valley Chern numbers at *K* and *K*′ take the opposite value ±1/2. At the interface formed by two materials with opposite valley Chern numbers, there are two gapless edge states protected by the valley Chern number. The edge states are also referred to as the kink states. There is no strict bulk topological invariant to illustrate the bulk boundary correspondence of the domain state in the valley Hall effect. However, it is proposed that the bulk-edge correspondence in the valley topological insulator may be understood from the Weyl physics in the synthetic higher dimensional space, which unveils the elusive valley band topology [87].

**Figure 3:**
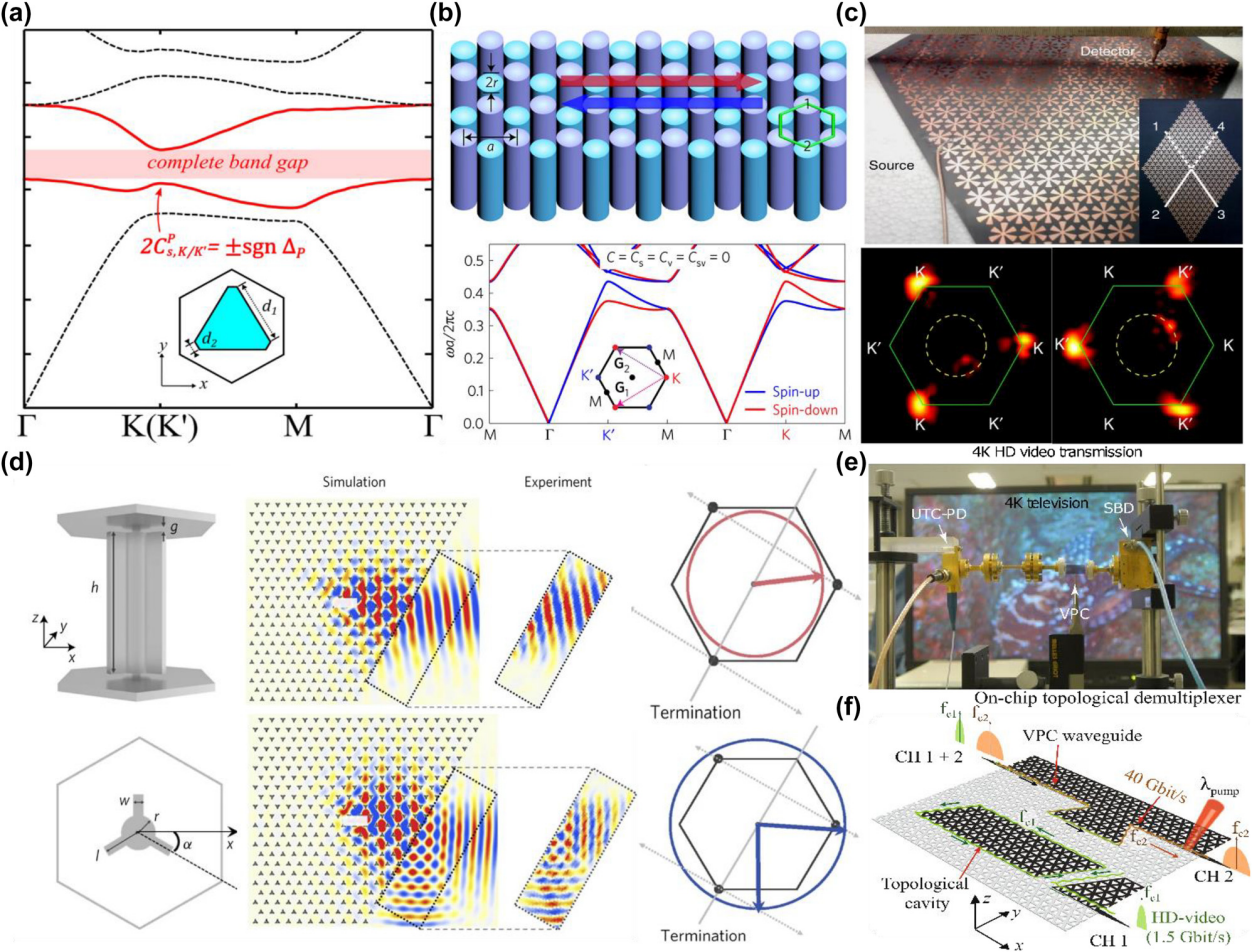
Photonic valley Hall systems. (a) Photonic band structure of the all Si triangular rod, the unit cell is shown in the inset [88]. (b) Schematic of the valley photonic crystal. Rods with different colors have opposite bianisotropy, which break the inversion symmetry. Lower inset: The energy dispersion for valley photonic crystal, where blue and red curves denote the energy band for the spin up and spin down, respectively [89]. (c) Direct observation of valley polarized kink state in the surface plasmon crystals [94]. (d) Topological protected refraction of kink state to the vacuum space [55]. (e) Robust terahertz communication based on topological kink state [95]. (f) Phototunable multi-channel on-chip terahertz communication based on topological photonic valley crystal [96].

T. Ma et al. first proposed the design of valley Hall photonic topological insulator in a hexagonal crystal [88]. A nontrivial bandgap protected by a nonzero valley Chern number emerges by changing the Si rods from a circular shape to a triangular shape, as shown in Figure 3a. In addition to the valley degree of freedom, J. W. Dong et al. take the spin into account [89]. The spin bulk bands are split at different valleys and achieve valley-selective net spin flow inside the bulk crystal, as shown in Figure 3b. As discussed in the previous section, bianisotropic metamaterials can be utilized to engineer the pseudospin *ψ* ^±^ = TE ± TM in photonic systems. Thus, a pseudospin photonic valley Hall system can be designed based on bianisotropic metamaterials [90–93]. The experimental observation of the valley-polarized kink state is achieved in the surface plasmon crystal [94], where it is easy to directly observe the topological kink state by near-field mapping. The experimental setup is shown in the upper panel of Figure 3c. As sketched in the inset of the upper panel of Figure 3c, the sample comprises 4 domains, whose upper and lower domains are filled with patterns A, and the left and right domains are filled with patterns B. The BA and AB type domain walls are denoted by the solid and dashed lines in the inset, respectively. By performing the spatial Fourier transformation of the experimentally scanned *Ez* component, the valley-polarized kink state is mapped to the *K* or *K*′ point in the first BZ as sketched in the lower panel of Figure 3c. Since the kink states are valley polarized, photonic valley crystals can also achieve topologically protected refraction from valley photonic crystals to the surrounding environment [88, 93]. The waveguide dispersions for the *TE* and *TM* waves are $k_{TE}=\sqrt{{\left(\omega /c\right)}^{2}-{\left(\pi /d\right)}^{2}}$ and *k*_TM_ = *ω*/*c*, respectively, where *ω* is the angular frequency. To maintain momentum conservation, the wavevector *k* in the free space should satisfy $k\cdot e_{\text{zig}}=K_{i}^{\prime }\cdot e_{\text{zig}}$ , where $K_{i}^{\prime }$ is the selected valley in the valley photonic crystal and *e*_zig_ is the vector parallel to zigzag termination. As graphically shown in Figure 3d, there is one solution for the TE mode but two solutions for the TM mode, which is in good agreement with the experimental and simulation results.

Topological photonic valley Hall systems have great potential in device applications, such as topological on chip communication [95, 96] and topological cavities [97–99]. Terahertz on chip communication was experimentally achieved by Y. Yang et al. [95]. A graphene-like lattice was designed, where each unit cell consists of two equilateral triangular holes with unequal side lengths. Silicon platform–based photonic valley Hall crystals have the merits of high density integration and low energy loss. The experimental setup is shown in Figure 3e. The experiment demonstrates the transmission of 4 K high-definition video, where the transfer rate is up to 11 Gbit/s at 0.335 THz. Recently, terahertz silicon-based phototunable on-chip devices were designed [96]. Broadband single channel communication with 160 GBit/s and 125 GBit/s data rates in straight and bent topological waveguides has been achieved. Furthermore, a phototunable on-chip topological demultiplexer was designed. As shown in Figure 3f, channel 1 + 2 can be demultiplexed into two well-isolated channels. The experiments demonstrate 1.5 GBit/s real-time HD video transfer in channel 1 and 40 GBit/s data transfer in channel 2 at the same time. Photoexcited channel 2 can turn off the transmission while having a negligible effect on channel 1.

## 3 Photonic topological semimetal

### 3.1 Weyl points and Dirac points

Topological semimetals are important constituents of 3D topological phases and have attracted considerable attention due to topologically protected surface states and novel transport phenomena [100], such as chiral anomaly [101, 102] and quantum oscillation [103]. The point, line, and surface band degeneracy correspond to Weyl (Dirac), nodal line, and nodal surface semimetals, respectively. The fundamental 3D degenerate band structure is the Weyl point, which features point degeneracies between two bands in the 3D momentum space. The Weyl point, acting like a relativistic fermionic particle, is considered as a monopole of Berry flux in 3D momentum space carrying a nonzero topological charge. The dispersion around the Weyl point is linear along all three directions, which can be described by *H*(*k*) = *v*(*q*_*x*_ *σ*_*x*_ + *q*_*y*_ *σ*_*y*_ + *q*_*z*_ *σ*_*z*_).

Different from the 2D Dirac cone, which can be easily removed by breaking *T* or parity inversion (*P*) symmetry, the single Weyl point is difficult to destroy because the Hamiltonian of the Weyl point contains all three Pauli matrices. The Weyl points must be created and annihilated in pairs with opposite topological charges guaranteed by the Nielsen–Ninomiya theorem. The remarkable topological property of Weyl points is the existence of surface states, well known as Fermi arcs, connecting pairwise Weyl points with opposite topological charges [104].

**Figure 4:**
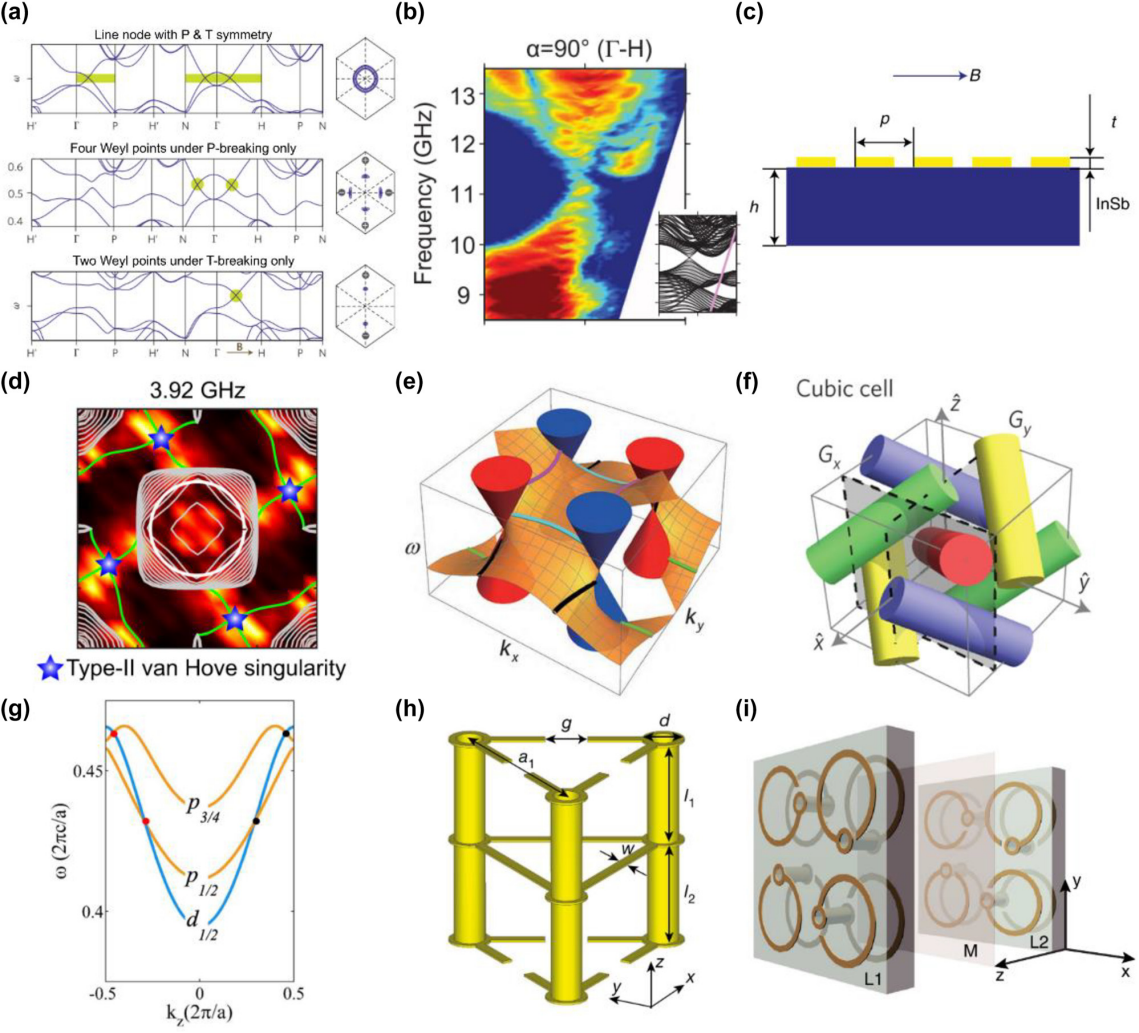
Point degeneracy of photonic topological semimetals. (a) The band structure of the line node and Weyl points in the DG photonic crystal [27]. (b) The observation of projected band structure in the DG photonic crystal with *P*-symmetry breaking [105]. (c) Schematic of magnetized semiconductor system for realizing *T*-symmetry breaking Weyl points [113]. (d) The surface iso-frequency contours with quadruple-helicoid topological surface and type-II van Hove singularity [130]. (e) Four ideal Weyl points with helicoid surface states [31]. (f) The unit cell of photonic Dirac topological semimetal with two glide reflection symmetry [135]. (g) The band structure of the type-II Dirac point formed by band inversion [141]. (h) The unit cell of Dirac metamaterial realizing in a photonic structure with C_3_ symmetry [136]. (i) Schematic of the Dirac metamaterial with electromagnetic duality symmetry [28].

Lu et al. first proposed a scheme for realizing Weyl semimetal in photonics [27]. The double-gyroid (DG) photonic crystal shows quadratic point degeneracy at the Brillouin center. In the DG photonic crystal protected by *T* and *P* symmetry, the quadratic point is lifted into ring degeneracy by applying perturbation. The nodal ring is topologically trivial, and Weyl points can be obtained by further breaking the *P* or *T* symmetry, as shown in Figure 4a. *T* maps a Weyl point at ***k*** to −***k*** with the same chirality, while *P* maps a Weyl point at ***k*** to −***k*** with the opposite chirality. In addition, the net chirality of Weyl points must be zero to neutralize the whole system. Therefore, the minimal number of Weyl points is two pairs in systems with only *P* symmetry breaking, while it is one pair in systems with only *T* symmetry breaking. Because the *T*-breaking system requires an external magnetic field and the magnetic response of existing optical materials is limited, it is difficult to observe the Weyl points by breaking the *T* symmetry. The first experimental realization of the Weyl point in a photonic system at microwave frequencies was achieved by breaking *P* [105]. The *P*-breaking DG photonic crystal is realized by adding an air sphere on only one of the gyroids. The dispersion of the bulk states measured by angle-resolved transmission presents a Weyl point along Γ − H, as shown in Figure 4b. Subsequently, E. Goi et al. present a novel route to realize Weyl points in bio-inspired gyroid photonic crystals coated with nanometric materials [106]. Other theoretical schemes for achieving Weyl points by breaking the *P* symmetry have also been proposed in photonic crystal superlattices [107] and chiral metamaterials [108, 109]. These schemes can stimulate further research on the experimental observation of Weyl points in the optical regime and lead to new potentials for the manipulation of electromagnetic and quantum information processing.

Several theoretical works for realizing *T*-breaking Weyl points have also been proposed in magnetized plasma [110], magnetic tetrahedral photonic crystals [111], and gyromagnetic metamaterials [112]. The magnetized plasma system has obvious advantages. The system does not involve complicated structural design and fabrication and can be reconfigured by adjusting the external field. The experimental observation was realized in a magnetized semiconductor-InSb, which behaves as a magnetized plasma for electromagnetic waves in the terahertz regime [113]. By applying a magnetic field parallel to the sample surface, Weyl points appear along the direction of the magnetic field. The Weyl point was constructed in the synthetic parameter space [*k*_*x*_, *k*_*y*_, *B*] instead of the [*k*_*x*_, *k*_*y*_, *k*_*z*_] space, as shown in Figure 4c. Very recently, a single pair of Weyl points was observed in gyromagnetic photonic crystal with *T*-breaking by applying magnetic field [29]. When adjusting the external magnetic field strength, this gyromagnetic photonic crystal will present three topological phases successively: topological trivial, Weyl semimetal, and topological nontrivial phases.

The Weyl point is dubbed as type-II Weyl point when the conical dispersion of the Weyl point is so strongly tilted that the group velocity signs of the crossing band are the same along one direction [114]. The type-II Weyl point possesses a conical Fermi surface and its physical properties are very different from the type-I Weyl point. Type-II Weyl points have numerous unique transport properties, including antichiral Landau levels and anisotropic chiral anomaly. Photonic type-II Weyl points have been realized in chiral metamaterials [115, 116], waveguides arrays [117], and twisted dielectric photonic crystals [118]. Furthermore, type-II Weyl points have also been observed in other classical systems such as acoustic crystal [119, 120] and circuit systems [121].

Weyl points carrying multiple topological charges higher than unity can exist in certain crystals with additional spatial symmetry [122]. The presence of both single and multiple Weyl points was observed in photonic crystals stacked by 2D honeycomb lattices with introducing appropriate interlayer couplings [123]. The double Weyl points are protected by *C*_3_ and *T* symmetries while triple Weyl points are protected by *C*_6_ symmetry. Subsequently, Weyl points with a topological charge of 2 were proposed in simple woodpile photonic crystals [124, 125]. The woodpile photonic crystal can fabricate down to the nanoscale, making them promising platforms to achieve Weyl point and relevant phenomena in the high-frequency regimes. Multiple Weyl points in woodpile photonic crystals fabricated by two-photon polymerization were observed in the mid-infrared regime [126]. Research found that multiple Weyl points can be obtained by merging unit-charge Weyl points, and the maximal topological charge of the twofold Weyl point is four [127–129]. Very recently, the Weyl point with the maximum topological charge was realized in a 3D photonic crystal with *C*_3,111_ symmetry [130]. The quadruple spiral Fermi arcs, which form two nonretractable loops in the surface BZ and the optical type II van Hove singularities, have been experimentally observed, as shown in Figure 4d.

Another type of Weyl point, which all Weyl points host the same frequency and are separated from the other bands, is dubbed ideal Weyl point [131, 132]. Such ideal Weyl points in a photonic crystal have qualitative advantages. For example, at Weyl point frequency, momentum degeneracy is extremely reduced to discrete momentum points. In addition, the linear dispersion near Weyl points gives rise to an effective refractive index, which can be either positive or negative. These exotic advantages may pave new avenues to realize novel optical devices and applications such as frequency selectivity, positive and negative refractive index, invisibility cloaking, and 3D imaging. The ideal Weyl point was experimentally realized in a microwave photonic crystal with saddle-shaped metallic coils [31], where the ideal Weyl system is protected by *D*_2*d*_ point symmetry and connected by helicoidal surface states, as shown in Figure 4e. Moreover, ideal photonic Weyl points with a charge of 2 and relevant long surface arcs have been observed in a chiral metamaterial in the microwave regime [133]. Compared with the woodpile photonic crystal that many trivial bands locate at the multi Weyl point frequency [126], the ideal photonic Weyl points all existed at the same energy frequency can further promote the development of Weyl physics and novel optical devices.

Dirac points are the fourfold degenerate points in 3D momentum space, representing the degeneracy of two Weyl points with opposite topological charges [100, 134]. When the symmetry of the system is broken, the Dirac points can transit into charged nodal lines, Weyl points, and open bandgap supporting gapless surface states [135, 136] or chiral hinge states [137, 138]. Dirac points are mainly divided into two categories according to their formation mechanism. Symmetry-enforced Dirac points are unavoidable results of the nonsymmorphic space group of the system. They are located at the high-symmetry points of the BZ and are robust against perturbations. Dirac points formed by band inversion are located at the generic moment of the axis of rotation symmetry and always appear in pairs. The symmetry-enforced Dirac points can be constructed in the photonic crystal with glide reflections and inversion, and its unit cell is shown in Figure 4f [135]. The Dirac points are predicted at the high-symmetry points of the BZ. The glide reflections also lead to exotic surface dispersion exhibiting quad-helicoid surface states, which has been experimentally demonstrated in acoustic crystals [139, 140].

Wang et al. proposed an all-dielectric photonic crystal with screw symmetry to construct the type-II Dirac point [141] by crossing the *p* and *d* bands, as shown in Figure 4g. The type-II Dirac points with screw crystalline symmetry can split into Weyl points when the space symmetry is reduced [142]. Based on band inversion, Dirac points can also be found in a hexagonal structure with *C*_3_ or *C*_6_ symmetry [136, 143]. The unit cell of the photonic crystal consisting of metallic split-ring resonators with *C*_3_ symmetry is depicted in Figure 4h [136]. The photonic band structure exhibits 3D Dirac points with a finely tuning lattice structure parameter. When the upper three split-ring resonators are removed, the photonic band structure opens a wide bandgap, and the system enters a 3D topological insulator phase. A similar 3D topological insulator phase can be realized in an all-dielectric crystal with broken mirror symmetry [71]. In photonic crystals, Dirac points can be achieved by not only spatial symmetries but also electromagnetic duality symmetry [28, 144]. Electromagnetic duality is an intrinsic property of the Maxwell equations in vacuum and can be utilized to realize a topological phase. Two symmetrically distributed Dirac points stabilized by electromagnetic duality symmetry have been experimentally demonstrated in a Dirac metamaterial [28]. The unit cell consists of two layers of metallic helical elements, as shown in Figure 4i. The double degenerate longitudinal modes and circularly polarized transverse modes can be generated by fine-tuning the structure parameter. Meanwhile, exotic spin-polarized topological surface states have been observed at the boundary between air and the Dirac metamaterial, which may have unique advantages for spin-multiplexed topological surface wave propagation.

### 3.2 Nodal lines and nodal surfaces

In addition to Weyl points and Dirac points, the band structure of semimetals has other forms, such as nodal lines [145, 146] and nodal surfaces [147], which have much richer topological properties. Like point degeneracy, line and surface degeneracy are protected by band topology and symmetries. By breaking symmetry, the nodal lines and surfaces can be partially or fully gapped and transform into Weyl semimetals, Dirac semimetals, or topological insulators. Due to the difference in degenerate dimension, nodal lines and nodal surfaces semimetal have more abundant surface states and interesting physical phenomena. According to the shape of the degeneracy, nodal lines can exhibit nodal rings [148], nodal chains [149], nodal links [150], and nodal knots [151], which have much richer topologically distinct possibilities. The nodal line possesses a key feature, that is, the Berry phase around the nodal line is *π*.

**Figure 5:**
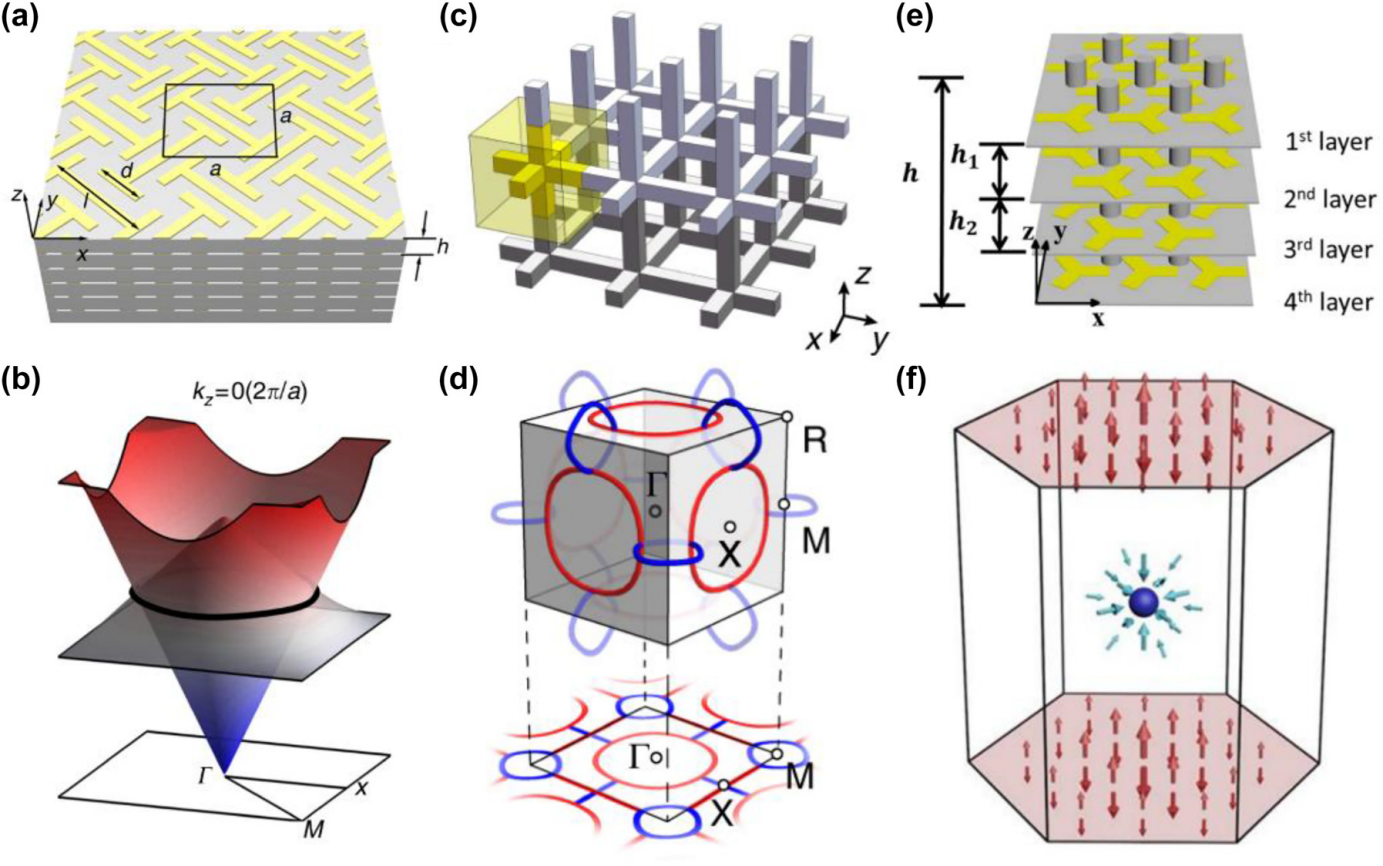
Line and surface degeneracy of photonic topological semimetals. (a) Schematic of the microwave metacrystal and (b) the band structure of the metacrystal with nodal ring [153]. (c) Illustration of the metallic-mesh photonic crystal and (d) nodal chains in the BZ [154]. (e) 3D model of the charged nodal surface structure and (f) the Berry flux represented by arrows in the vicinity of charged nodal surface and Weyl point [157].

An hourglass nodal line with an hourglass-shaped band dispersion protected by three glide mirror symmetries and *C*_4_ symmetry was first observed in photonic metacrystal, which is robust against symmetry preserving perturbations [152]. Recently, a scheme for nodal ring has been proposed in DG photonic crystal [27]. The photonic nodal rings were experimentally demonstrated in a microwave cut-wire metacrystal with glide symmetry, whose unit cell is shown in Figure 5a [153]. The relevant drumhead surface states in the nodal ring semimetal can be observed by near-field scanning. The 3D band structure presents a ring degeneracy, which can transform into two Weyl points under a static magnetic field, as plotted in Figure 5b. In addition to the unique topological characteristics, the nodal degeneracy and donut-shaped equi-frequency surface lead to other interesting phenomena such as spontaneous emission, resonant scattering, and black-body radiation. Nodal rings chained together can generate nodal chains [149], where the Berry phase around the chain point is zero. Nodal chains were experimentally demonstrated in a metallic mesh photonic crystal, as shown in Figure 5c [154]. The nodal chains go across the entire BZ, and the relevant structures are depicted in Figure 5d. The nodal chains protected by mirror symmetry will be lifted when the mirror symmetry is broken.

The band dispersion of the nodal surface shows a 2D band degeneracy surface in momentum space, which exists in the system with nonsymmorphic symmetry and *T* symmetry. A photonic nodal surface carrying a nonzero topological charge was proposed in a helix structure with twofold screw symmetry and *T* symmetry. Nodal surfaces with nonzero topological charge in the classical system were first realized in acoustic crystals [155, 156]. Subsequently, the photonic counterpart was experimentally observed in an electromagnetic metacrystal at microwave frequency [157]. The metacrystal possessing twofold screw symmetry can be fabricated by printed circuit board, as shown in Figure 5e [157]. The nodal surface carries a topological charge of +2 at *k*_*z*_ = *π*/*h*, and a Weyl point hosts a topological charge of −2 at *k*_*z*_ = 0 in the BZ, which are viewed as the source of the Berry flux, as depicted in Figure 5f. There are two surface states connecting the Weyl point and the nodal surface, rotating around the Weyl point in a counterclockwise manner as the frequency increases. The robust unidirectional surface states have great potential on photonic devices, which may find application in the construction of one-way waveguides, optical isolators, and the beam splitter.

### 3.3 Applications of topological semimetals

Topological semimetals possess topologically protected surface states and novel topological transport phenomena, which bring new potential to the manipulation of electromagnetic waves such as one-way waveguide [158], frequency selectivity [132], topological self-collimation [133], optical tweezers [159], cloaking [160], and Veselago imaging [161]. The presence of chiral zeroth Landau levels in Dirac and Weyl systems is the physical origin of chiral transport. The chiral zeroth Landau level, which is a one-way propagative bulk mode, can be applied for robust transport of photons in the bulk medium [101]. Next, we give a brief introduction to several potential applications of semimetals, such as the generation and manipulation of vortex beams [162], topological negative refraction [163], and surface emitting lasers [164].

**Figure 6:**
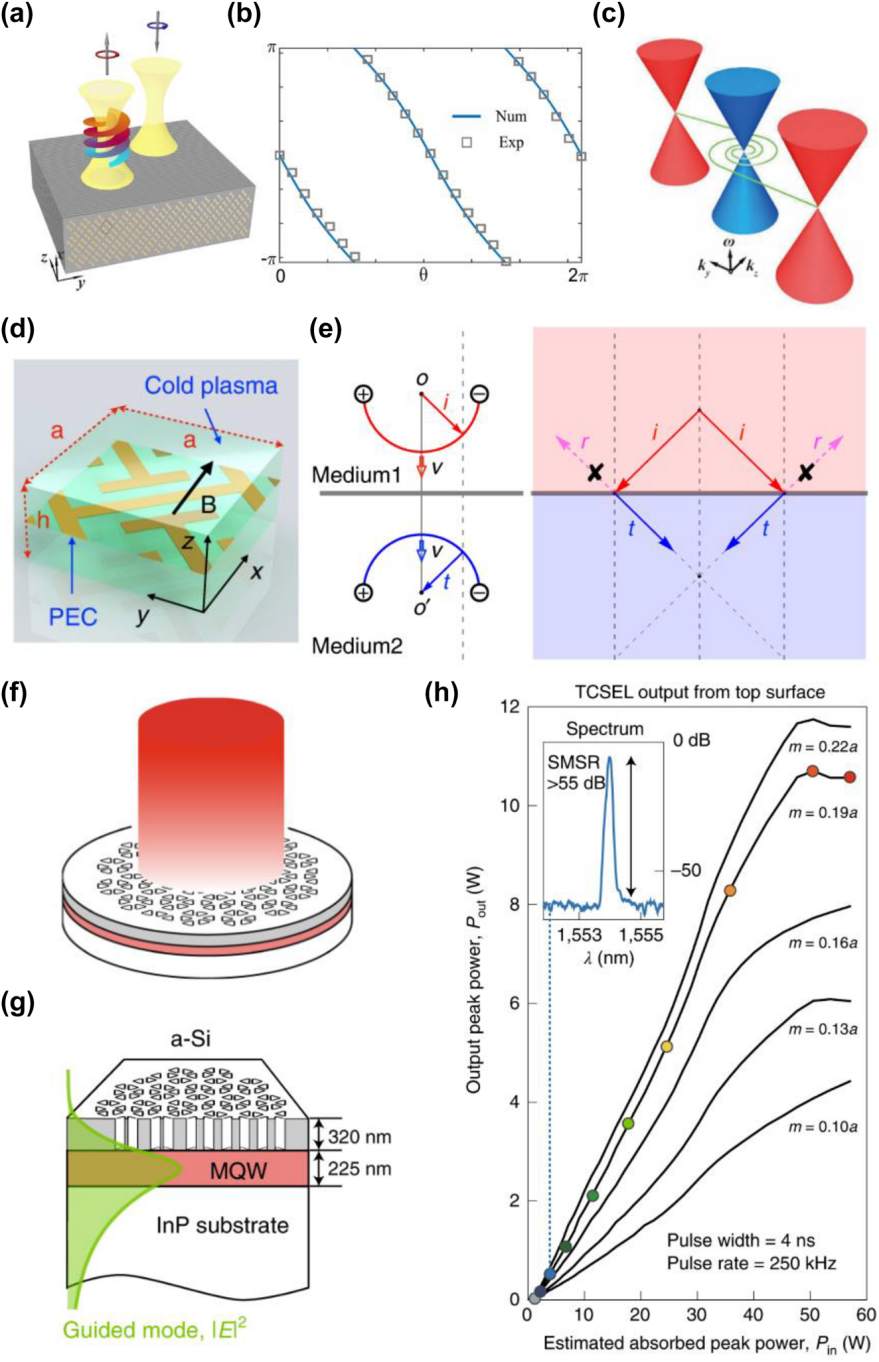
Applications of photonic topological semimetals. (a) The sketch of the generation of vortex beam, (b) phase of the reflected vortex beam, and (c) spiral surface states with projected Weyl nodes in the surface BZ [162]. (d) Unit cell of the ideal photonic Weyl metamaterial and (e) all-angle reflectionless negative refraction [163]. (f) Schematic of topological-cavity surface-emitting laser [168]. (g) Structure of topological cavity surface-emitting laser, where the vertical-mode is denoted by green line [168]. (h) Light-in and light-out curves of topological cavity surface-emitting laser with different modulation amplitudes *m* [168].

Cheng et al. proposed alignment-free phase plates for the generation of vortical phase and vortex beams using the reflection property of Weyl metamaterials [162]. The topological properties of photonic semimetals can be revealed not only by the Fermi arc but also by the vortical phase profile in the spin-polarized electromagnetic wave reflection. When a beam with spin angular momentum is incident on the Weyl metamaterial, the reflected beam will carry additional orbital and opposite spin angular momentum, as shown in Figure 6a. Due to the translation invariance of Weyl metamaterials, the incident beam does not need to be aligned at a specific position on the metamaterial. The topological classification of the Hamiltonian and the scattering matrix are equivalent, and the scattering matrix around the Weyl point is given as:

(4) $$\hat{s}=e^{-i\theta }\left[\begin{array}{cc} \cos\theta & \sin\theta \\ \sin\theta & -\cos\theta \end{array}\right]$$

where *θ* is the angle between the reflection measurement plane and the *z*-axis. The scattering matrix operated on the right-handed circular polarization state $\left|R\right\rangle$ is converted to the light-handed circular polarization state $\left|L\right\rangle$ with an extra 2*θ* phase but the conversion from $\left|L\right\rangle$ to $\left|R\right\rangle$ without extra phase. Therefore, an incident Gaussian beam with polarization state $\left|R\right\rangle$ can be reflected into a vortex beam with an orbital angular momentum of 2. Both numerical simulation and experimental measurements show that the reflected beam with right-handed elliptical incident light has a winding reflection phase of 4*π*, as given in Figure 6b. The nontrivial reflection phases lead to spiral Fermi arcs that connect the projected Weyl points in the momentum space, as shown in Figure 6c. In addition, vector or vortex beams can also be generated through the interaction of light with photonic Dirac metamaterials [144]. In the momentum space near the Dirac point, an incident beam with right-circularly polarized or left-circularly polarized light acquires a 2*π* or −2*π* reflection phase, leading to the generation of a vortex beam in reflection. When incident Gaussian beams are in TE and TM polarization states, angular and radial vector beams can be generated in reflection. The topological property of the semimetals provides a novel method for generating vector and vortex beams. It is a much more convenient approach than the traditional method based on the phase element of correlation space variation.

Topological negative refraction was proposed by Liu et al. in a Weyl phononic crystal [165]. Recently, all-angle reflectionless negative refraction was experimentally achieved in an ideal photonic Weyl semimetal [163]. As shown in Figure 6d, the Weyl semimetal is constructed by embedding a periodic metallic structure with nonsymmorphic symmetry into magnetized cold plasma, where a pair of ideal Weyl points can be obtained by an external magnetic field. The Fermi arcs form a semicircle at the surface by the carefully chosen surrounding medium, as shown in Figure 6e. In contrast to conventional negative refraction, where reflection is generally allowed, reflection is forbidden in topological negative refraction due to the open Fermi arc. Therefore, topological negative refraction can theoretically collect all incident waves and be used for robust negative index flat lens.

On the other hand, a Dirac-vortex topological cavity has been proposed based on a 2D Dirac point. Dirac-vortex cavities experimentally realized in a silicon-on-insulator platform have scalable mode areas, arbitrary mode degeneracies, vector-beam vertical emission, and compatibility with high-index substrates [166]. Dirac-vortex fiber has been constructed by the mid-gap defect mode of the Dirac-vortex gap, where the number of guiding modes equals the winding number of the spatial vortex [167]. Recently, a Dirac-vortex cavity was used to realize a surface-emitting laser that exhibits 10 W peak power, sub −1° divergence angle, and 60 dB side-mode suppression, as shown in Figure 6f [168]. The structure of the topological cavity surface-emitting laser is shown in Figure 6g, where the topological cavity is formed by placing an amorphous silicon photonic crystal layer on a waveguide layer with multiple quantum wells on the InP substrate. When the output power is below 1 W, a side-mode suppression ratio of 55 dB and a linewidth of 0.03 nm are obtained, as shown in the inset of Figure 6h. The light-in and light-out curves show a continuing increase in slope with modulation amplitude *m*, ranging from 0.10*a* to 0.22*a*, as plotted in Figure 6h. The output power has a remarkably high peak power of 10 W at 1550 nm, hosting a higher output power compared to a commercial 1550 nm distributed feedback laser. Dirac-vortex cavities can also be readily integrated with topological waveguides to explore the potential of topological photonic circuitry and provide a new method for the design of on-chip photonic devices.

## 4 Non-Hermitian topological photonics

Optical topology has found great success in the closed and lossless systems discussed above, where the physics is characterized by the Hermitian operator. One of the key assumptions in quantum mechanics requires the Hermiticity of physical observables, which ensures probability conservation. As ensured by Hermitian operators, Hermitian systems host not only the realness of the eigenvalues but also the orthogonality of a complete set of bases. However, most physical systems are generically non-Hermitian, arising from open boundaries or gains/losses, and do not follow this rule. Developed from an overall growth or decay of the norm for quantum states in quantum field theory [169, 170], non-Hermitian systems are characterized by complex eigenvalues with a nonorthogonal basis. Non-Hermitian systems in quantum mechanics are helpful to explore the loss, finite lifetime, particle decay, and scattering problems, but they always follow the underlying assumption in quantum mechanics that observables of physical systems must be real quantities.

A significant change in our cognition of non-Hermitian occurred when Bender and Boettcher realized that a special class of non-Hermitian Hamiltonians hosts entirely real eigenvalues as long as they obey parity-time (*PT*) symmetry [171]. The relevant non-Hermitian systems have been found to exist in various physical systems, including solid-state materials, photonics [172–176], phononics [177–182], mechanics [183, 184], and cold atoms [185, 186]. The introduction of *PT* symmetry has special significance for optical systems, which means that the gain and loss that reduce the laser performance will be used as the non-Hermitian parts of the operators to achieve a new photonic state of matter and explore new physical phenomena. In addition, *PT* symmetry is readily established by judiciously distributing the gain and loss in the photonic lattice, thus optical systems have developed into an important platform for non-Hermitian research. *PT* symmetric photonics lead to counterintuitive phenomena, such as nonreciprocal light propagation [187, 188] and orbital angular momentum microlasers [189].

**Figure 7:**
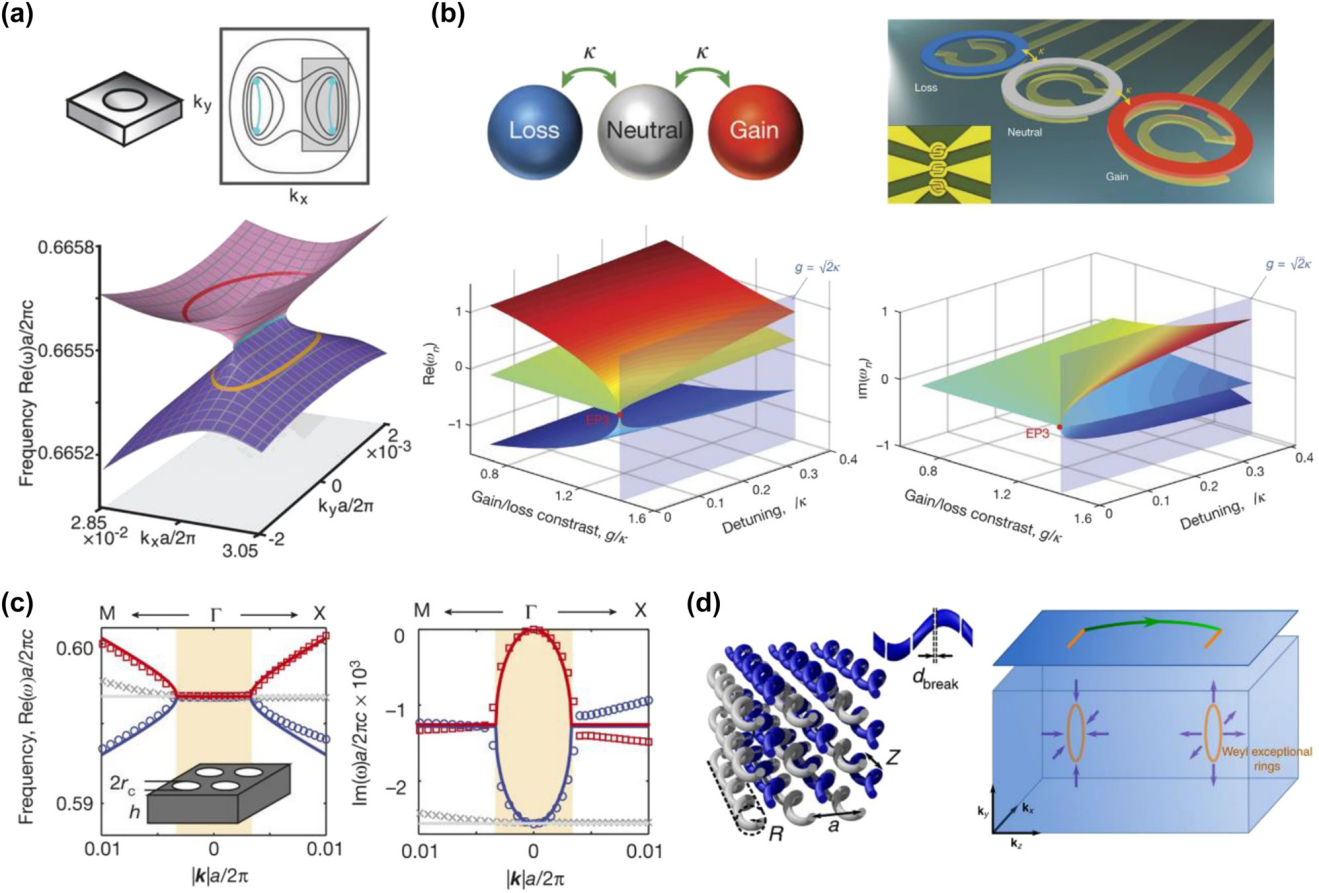
Exceptional degeneracy in non-Hermitian systems. (a) A pair of EPs in a 2D periodic photonic crystal (top) [190]. Band structures in *k*_*x*_ − *k*_*y*_ plane, where Fermi arc connects a pair of EPs (bottom). (b) Third-order EP in PT symmetric coupled cavity systems [192] (top). Band structures with complex eigenfrequencies in the parameter space, exhibiting a third-order (bottom). (c) Exceptional rings realized in a photonic crystal slab, where complex eigenvalues exhibit exceptional rings in the 2D k-space [196]. (d) Bipartite helical waveguide array, where breaks are added to one of the sublattices to remove the Hermiticity [209]. The projection of a pair of Weyl exceptional rings are connected by surface states.

In the last few years, due to the development of topological photonics, the field has embarked on new topological states and novel topological phenomena in non-Hermitian systems. One of the most typical examples is the study of non-Hermitian degeneracies. For a *PT* symmetric Hamiltonian, when the non-Hermitian parameter exceeds a certain critical threshold, the entirely real eigenvalues of the system become complex eigenvalues with a phase transition from a *PT* unbroken phase to a *PT* broken phase, arising from spontaneous symmetry breaking. The phase transition presents a type of special degeneracy, which requires a new topological invariant description, dubbed as exceptional points (EPs) [190]. EPs have been explored in topological photonics with the aim of exploring the relationship between symmetries and topological phases in non-Hermitian systems. Many studies have theoretically and experimentally explored EPs with topological properties that go beyond the known Hermitian classifications. Recently, research has found that a pair of EPs can be obtained by splitting a Dirac point when radiation loss is added to a 2D periodic photonic crystal [190], as shown in Figure 7a. The non-Hermitian effects are characterized by a bulk Fermi arc connecting this pair of EPs. The bulk Fermi arc, as an open-ended isofrequency contour, resides at one frequency in the bulk dispersion and originates from non-Hermiticity, distinct from the known surface Fermi arc in 3D topological semimetals. By observing the far-field polarization around the Fermi arc, it can be proven experimentally that EP carries a topological charge of ±1/2. These special non-Hermitian effects are rooted in a distinct double Riemann sheet topology in the complex band structure generated by this EP pair. Further research found that the bifurcation properties of EPs can enhance the sensitivity and have wide application prospects in various sensing arrangements [191]. In addition to the EPs discussed above, higher-order EPs (greater than second order) have been brought into focus due to their topological properties of greater sensitivity. Recently, higher-order EPs were observed in a *PT* symmetric ternary micro-ring system with equidistantly spaced cavities, as shown in Figure 7b [192]. The third-order EP is constructed by three resonators with resonance wavelength tuning and thermal perturbations, where one cavity experiencing gain and another experiencing an equal amount of loss are separated by a neutral cavity. Similar to the degeneracy in Hermitian systems, degeneracy in 2D non-Hermitian systems can also exhibit richer geometric features, such as exceptional rings and exceptional surfaces [37, 193]. It has been found that a ring of exceptional points can be constructed by adding certain non-Hermitian perturbations to the Dirac cones [194, 195]. The theoretical proposes are realized in a photonic crystal slab [196], where complex eigenvalues with exceptional rings are supported in the 2D k-space, as shown in Figure 7c. So far, the research on non-Hermitian degeneracies across diversified photonic platforms has become an attractive topic, which has witnessed a series of amazing topological properties, such as topological chirality [197–199], unidirectional invisibility [172, 187, 200], laser mode selectivity [201, 202], lasers with reversed pump dependence [203], and extraordinary sensitivities [192, 204], [205], [206]. In the last few years, non-Hermiticity has been explored in 3D topological materials, including Weyl semimetals. When a non-Hermitian perturbation is added to a Weyl semimetal, the Weyl point will split into a ring of EPs, dubbed as the Weyl exceptional ring, where the Berry charge remains real, quantized and the same charge as the original Weyl point [207, 208]. Recently, a Weyl exceptional ring was experimentally realized in a 3D photonic lattice composed of an evanescently coupled helical waveguide array [209]. The coupled helical waveguide ensures the formation of Weyl points, meanwhile breaks are added to one of the sublattices to break the parity symmetry of the system. The Weyl exceptional rings, rooted in the splitting of the Weyl points by non-Hermiticity, are shown in Figure 7d, where the Fermi arc surface states connect the projections of the Weyl exceptional rings.

**Figure 8:**
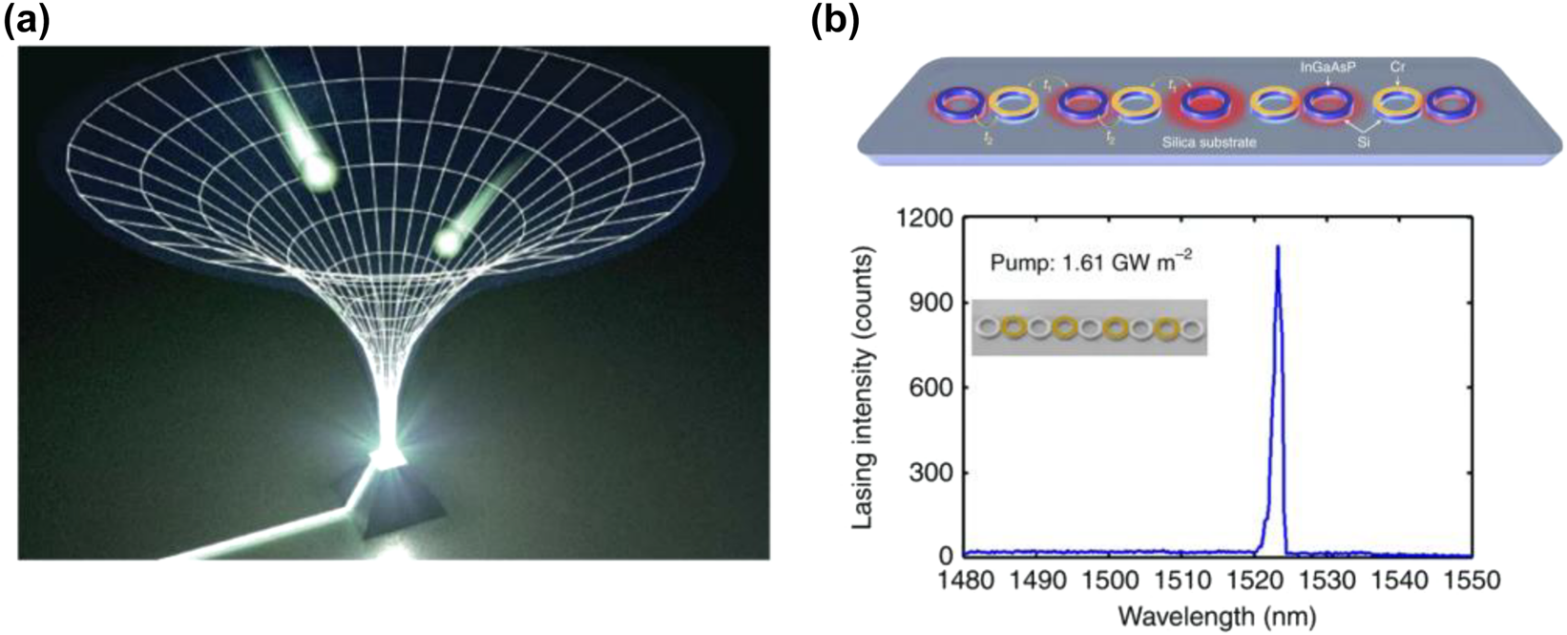
Applications of non-Hermitian topological effect. (a) Topological funneling of light, arising from non-Hermitian skin effects [210]. (b) On-chip hybrid silicon microlaser [215].

The topological properties of the systems, rooted in the bulk degeneracies with nonzero topological invariants, are represented by the appearance of boundary states around the topological materials. It is well known as bulk-edge correspondence, which reveals the relationship between the topologically protected boundary states and the bulk topological invariants in Hermitian systems. However, the introduction of a non-Hermitian perturbation exhibits not only exceptional degeneracies with anomalous topological winding numbers but also breakdowns of bulk-edge correspondence. The non-Hermitian skin effect [210–214], where the bulk modes with open boundaries are localized at the boundaries, challenges the existing Bloch band theory and urges us to re-examine the topological theory in non-Hermitian systems is evidence of breaking the bulk-edge correspondence in non-Hermitian topological systems. Recently, the non-Hermitian skin effect has been experimentally realized in a non-Hermitian photonic lattice by tailoring the anisotropy of the nearest-neighbor coupling [210]. The hallmark of the non-Hermitian skin effect is observed, showing an exponential localization of all modes at the interface. Based on this unexpected effect, the researchers demonstrated a highly efficient funnel for light, in which any light field within the lattice would move toward the funnel (interface), irrespective of its shape and input position, as shown in Figure 8a.

There is presently an ongoing effort to promote the application of topological properties to non-Hermitian systems, one of which is an important focus on topological lasers. Gain and loss are common in lasers, but they are generally unwelcome and require the use of amplification to overcome optical loss. On the other hand, if the non-Hermitian topological properties constructed by gain and loss can be transplanted into lasing systems, the performance of the laser will be revolutionized. So far, considerable effort has been made to overcome the collision between topological robustness and the other physical considerations in lasers. Recently, an on-chip hybrid silicon micro-laser was proposed based on the strategic combination of non-Hermitian and topological symmetries [215], as shown in Figure 8b. The laser supports a protected zero mode, where robust single-mode laser action prevails even in the presence of perturbations. Meanwhile, another research reported an observation of lasing topological edge states in 1D Su–Schrieffer–Heeger active micro-ring resonator arrays, which is nonlinear and highly non-Hermitian [216]. In addition, EPs in non-Hermitian systems are also utilized to enrich and improve laser performance. The frequency of EP is designed to at or very near the real axis, which is consistent with resonant frequency at the onset of laser emission, resulting in a number of lasing applications, such as, single-mode lasing [201, 202, 217, 218], chiral lasing [199], laser linewidth broadening [219, 220], loss-induced laser revival [221], and lasing/perfect absorption switching [221]. The introduction of non-Hermitian topology is also an effective method to control the light, which has initiated many exotic effects and applications, including light steering in a photonic integrated circuit [222], optical nonreciprocity in coupled optical resonators [187], and optical isolators on a chip [222]. These results open the way to understanding the relationship between non-Hermiticity and topology in active systems. There is no doubt that non-Hermitian topological photonics, arising as a new field, is emerging from many novel topological phenomena and bright application prospects.

## 5 Conclusion and outlook

Although the concept of topology was transplanted from condensed matter systems to optical systems in only a few decades, topological photonics has made amazing progress. Considerable efforts have been made to break through the limitations between the bosonic and fermionic nature of photons and electrons. To date, great success has been achieved in the exploration and observation of novel photonic topological phases and phenomena. Hosting a relatively clean band structure and flexible designability, optical systems have gradually developed into a unique platform for topological research and led to the development of topological physics in special fields, including nonlinear, non-Hermitian, and non-Abelian topology. On the other hand, the concept of topology in turn has profoundly influenced the development of the optical field, and the relevant robustness has brought great changes in the design and integration of novel optical devices. Further advances in fundamental research and device design in topological photonics may unleash the potential for applications, which may radiate to other systems, including electrons, phonons, cold atoms, and mechanical waves. We believe that the development of photonic topological semimetals will continue to promote wider areas, such as communication and quantum computation, and light the pathway toward a brighter future.
